# Impact of a tailored program on the implementation of evidence-based recommendations for multimorbid patients with polypharmacy in primary care practices—results of a cluster-randomized controlled trial

**DOI:** 10.1186/s13012-016-0535-y

**Published:** 2017-01-13

**Authors:** Cornelia Jäger, Tobias Freund, Jost Steinhäuser, Christian Stock, Johannes Krisam, Petra Kaufmann-Kolle, Michel Wensing, Joachim Szecsenyi

**Affiliations:** 1 Department of General Practice and Health Services Research, University Hospital Heidelberg, Im Neuenheimer Feld 130.3, Turm West, 4.OG, 69120 Heidelberg, Germany; 2 Institute of Family Medicine, UniversityHospital Schleswig-Holstein, Campus Lübeck, Ratzburger Allee 160, Haus 50, 23538 Lübeck, Germany; 3 Department of Medical Biometry, Institute of Medical Biometry and Informatics, University Hospital Heidelberg, Im Neuenheimer Feld 130.3, Turm West, 12.OG, 69120 Heidelberg, Germany; 4 Institute for Applied Quality Improvement and Research in Health Care GmbH, (AQUA-Institute), Maschmühlenweg 8-10, 37073 Göttingen, Germany

**Keywords:** Multimorbidity, Polypharmacy, Tailoring, Randomized controlled trial, Medication list, Medication review, Communication, Potentially inappropriate medication

## Abstract

**Background:**

Multimorbid patients receiving polypharmacy represent a growing population at high risk for negative health outcomes. Tailoring is an approach of systematic intervention development taking account of previously identified determinants of practice. The aim of this study was to assess the effect of a tailored program to improve the implementation of three important processes of care for this patient group: (a) structured medication counseling including brown bag reviews, (b) the use of medication lists, and (c) structured medication reviews to reduce potentially inappropriate medication.

**Methods:**

We conducted a cluster-randomized controlled trial with a follow-up time of 9 months. Participants were general practitioners (GPs) organized in quality circles and participating in a GP-centered care contract of a German health insurance. Patients aged >50 years, suffering from at least 3 chronic diseases, receiving more than 4 drugs, and being at high risk for medication-related events according to the assessment of the treating GP were enrolled. The tailored program consisted of a workshop for GPs and health care assistants, educational materials and reminders for patients, and the elaboration of implementation action plans. The primary outcome was the change in the degree of implementation between baseline and follow-up, measured by a summary score of 10 indicators. The indicators were based on structured surveys with patients and GPs.

**Results:**

We analyzed the data of 21 GPs (10 - intervention group, 11 - control group) and 273 patients (130 - intervention group, 143 - control group). The increase in the degree of implementation was 4.2 percentage points (95% confidence interval: −0.3, 8.6) higher in the intervention group compared to the control group (*p* = 0.1). Two of the 10 indicators were significantly improved in the intervention group: medication counseling (*p* = 0.017) and brown bag review (*p* = 0.012). Secondary outcomes showed an effect on patients’ self-reported use of medication lists when buying drugs in the pharmacy (*p* = 0.03).

**Conclusions:**

The tailored program may improve implementation of medication counseling and brown bag review whereas the use of medication lists and medication reviews did not improve. No effect of the tailored program on the combined primary outcome could be substantiated. Due to limitations of the study, results have to be interpreted carefully. The factors facilitating and hindering successful implementation will be examined in a comprehensive process evaluation.

**Trial registration number:**

ISRCTN34664024, assigned 14/08/2013

## Background

### Deficiencies in the care of multimorbid patients with polypharmacy

Patients suffering from multiple chronic conditions and being treated with polypharmacy (commonly defined as permanent intake of more than four drugs) [1] represent a constantly growing population [2] at high risk for preventable adverse drug reactions (ADR) [3], potentially avoidable hospital admissions [4] and preventable deaths [5]. Implementation of evidence-based recommendations for drug treatment is a challenge in patients with polypharmacy. Reasons for the high prevalence of preventable negative health outcomes in this patient group can be found in different areas of care:

Suboptimal *communication between physicians and patients* about medication-related issues in both inpatient and outpatient settings [6, 7] can cause medication errors due to inappropriate prescribing, low adherence to, or wrong application of a prescribed medicine [7, 8].

*Prescribing and monitoring errors* are common in primary and hospital care. They may lead to a potentially inappropriate medication (PIM), which can be determined by implicit criteria, such as the Medication Appropriateness Index (MAI) [9] and explicit criteria, such as the PRISCUS list, a German adaption of the Beers criteria [10]. Medication errors according to implicit criteria appear in 5% of all prescriptions [11]. The PIM prescription rate in the elderly according to the PRISCUS list in Germany is relatively stable around 23% with only a small decrease within the past years [12, 13]. Patients taking PIM are at higher risk for ADR [14] and hospital admission [15].

*Insufficient documentation and exchange* of medication-related information between health care professionals are a potential cause of prescribing errors and ADR. Since there is no established electronic system for data exchange between different health care providers in Germany [16], to date the written, paper-based medication list of each individual patient is the most important document for medication-related information. Yet, deficiencies concerning the quality and availability of medication lists are well known: In Germany, only 25–50% of patients with polypharmacy have a medication list [17]. Several studies showed discrepancies between the documented and actually taken medication in about 75% of the cases [18–20], with 25% of those discrepancies being considered potentially harmful [21]. Due to lacking standardization, important information is frequently lacking or in case of handwritten medication lists not readable [22].

### The German health system

Hence, optimal care for multimorbid patients with polypharmacy requires information sharing between multiple prescribers, input of pharmacological knowledge into clinical decision making, continuous monitoring, and counseling of patients. This is particularly challenging for general practitioners (GPs) who act as main providers and coordinators of care for most adults with chronic diseases. Germany has no formal gate-keeping system in ambulatory care [23], and the GP is not necessarily the central care provider, so that patients’ medication regimens may be adapted by various physicians without communicating with a GP. As an attempt to strengthen the coordinating role of GPs, some German health insurances offer “GP-centered care contracts (HzV)” [24]. For some HzV care contracts, regular participation in “quality circles” (QC) [25] is obligatory for GPs. QC comprise educational small group meetings of GPs of one geographical region and written feedback on their individual practice patterns and prescribing behavior on the basis of claims data.

### Evidence-based care

An increasing number of studies evaluate strategies to improve appropriate use of polypharmacy especially in older patients. Few studies have been done in Germany and difficulties of providing high-quality evidence in this field due to methodological challenges have been described [26]. So, it is still uncertain which strategies are most effective [27]. For the study “Implementation of recommendations for polypharmacy in multimorbid patients (PomP),” we identified three core recommendations on drug management in polypharmacy patients from the research literature which are also recommended in a German guideline for multi-medication in primary care [28]. They were chosen out of a range of recommendations, because they focus—as described above—on different aspects of care with substantiated deficiencies, namely communication, prescribing, and documentation:

- *Recommendation 1 on communication: structured medication counseling (SMC):* All patients with polypharmacy and additional risk factors for medication problems should receive SMC at least once per year. Beside medication-related information, SMC comprises a complete inventory of the actually taken medication (so called “brown bag review”) and the assessment of patient adherence and possible application problems. A separate appointment should be planned for SMC [29]. There is evidence that SMC increases patient satisfaction with health care [30] and adherence and reduce ADR and hospitalizations [8]. It has been shown that better physician-patient communication leads to better health outcomes [31, 32].
- *Recommendation 2 on documentation: consequent use of medication lists*: All patients with polypharmacy should take along an updated, complete, and comprehensible medication list, concordant with the template of the Drug Commission of the German Medical Association [14]. There is consensus that medication lists are an important and useful document for HCP as well as for patients [33, 34], which is emphasized by the fact that the patients’ right to receive a complete medication list has been regularized by the German E-Health law enacted in 2015 (German Social Security Code 5, §31). It is plausible that medication lists have a positive influence on health outcomes.
- *Recommendation 3 on prescribing: medication reviews to reduce PIM:* Physicians should review the medication regimens of patients with polypharmacy systematically with the aid of tools, such as the PRISCUS list [10] or the MAI [9]. Both tools integrate a substantial body of knowledge on drug treatment. There is evidence that systematic medication reviews reduce emergency department contacts at least in hospital settings [35].

### Tailoring

Implementation of evidence-based practice in health care is often hindered by specific barriers or facilitated by enablers, also referred to as “determinants of practice.” Tailored programs are programs explicitly designed to address such previously identified determinants [36]. This study is part of the “Tailoring Interventions for Chronic Diseases” (TICD) project [37], during which five tailored programs have been developed and evaluated in randomized controlled trials according to a coordinated research plan [38–42]. The concept of tailoring used in TICD has been described elsewhere [43].

The aim of the PomP study was to improve the implementation of three core recommendations in primary care practices by a tailored program.

## Methods

### Trial design

The aim of the PomP study was to assess the effectiveness of a tailored program to improve the implementation of three core recommendations for medication management in primary care practices. The primary outcome was the difference in the degree of implementation between baseline and follow-up, measured by a summary score based on 10 indicators. The study design was a cluster-randomized controlled trial with QCs as unit of randomization. Follow-up time was 9 months.

### Participants and setting

GPs participating in a GP-centered care contract of one large German health insurance (HzV AOK Baden-Wuerttemberg) and organized in QCs were recruited. For this purpose, the moderators of the QCs in one geographical region in South Germany were contacted. GPs agreeing to participate received a de-identified list of patients (based on analyses of insurance claim data) meeting the following eligibility criteria:

- Patients older than 50 years
- Enrolment into the HzV AOK Baden-Wuerttemberg care contract
- Prescriptions for more than four different drugs in at least 2 quarters of the preceding year
- Diagnosis of at least three chronic conditions based on a previously published diagnosis list with a total of 42 diagnosis groups [44]

The recruitment of GPs and the data management of insurance claims data was done by the Institute for Applied Quality Improvement and Research in Health Care (AQUA Institute).

GPs were asked to assess the risk of medication problems (e.g., due to non-adherence or prior hospitalizations due to ADR) of these patients and to enroll up to 25 high risk patients.

Exclusion criteria for GPs were participation in another study focusing on multimorbidity or polypharmacy during the previous year. Exclusion criteria for patients were a cognitive or clinical status which hindered active participation in the study.

### Intervention

**Fig. 1:**
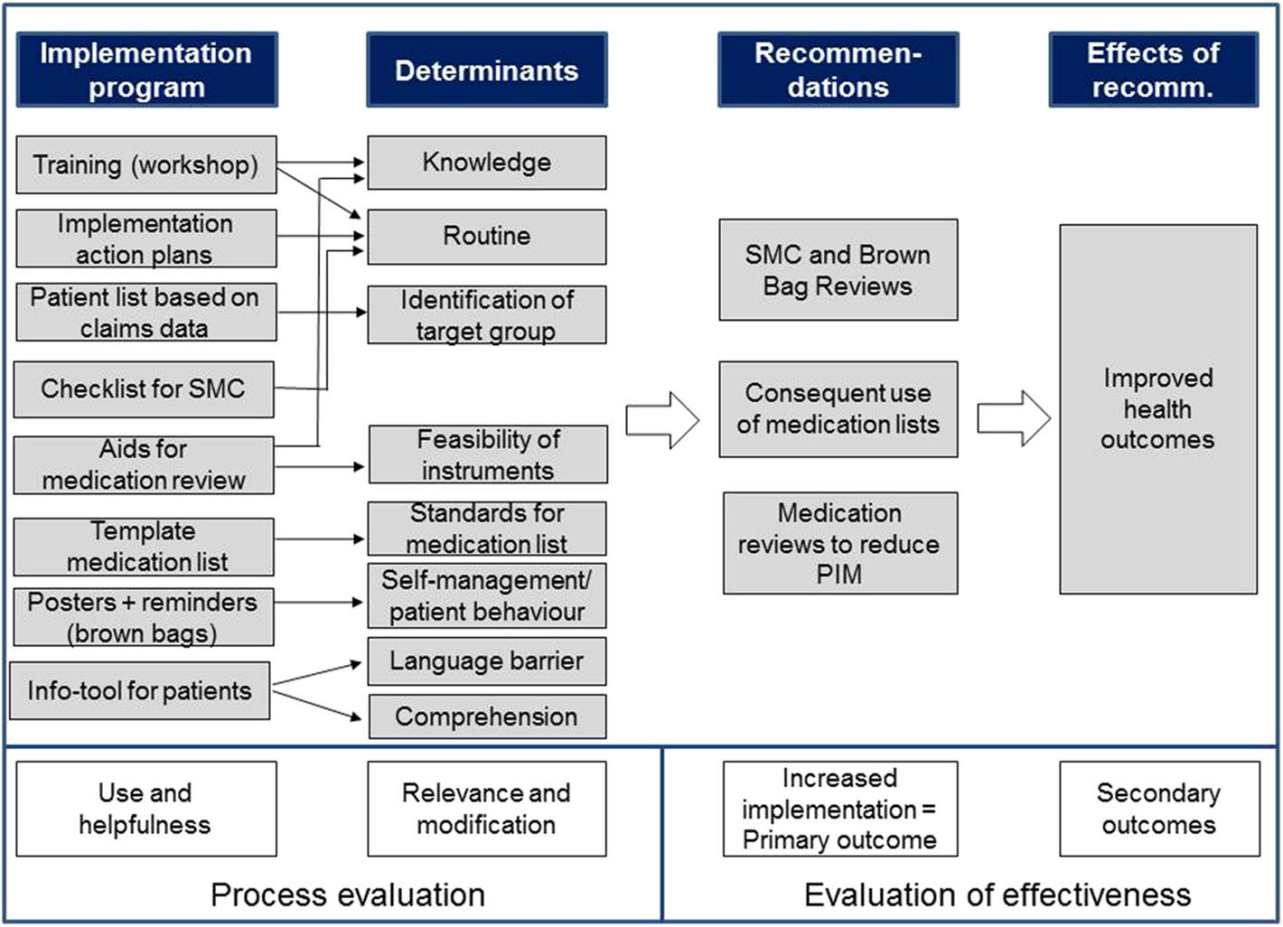
Logic model of the PomP intervention

The tailored program of the PomP study was developed based on previously identified determinants and strategies. The “logic model” illustrates the assumed mechanism of the tailored program (compare Fig. 1) which consisted of three major elements:

- *Training and resources for GPs and MAs*: During a four hour workshop for practice teams, GPs and MAs discussed potential barriers and solutions for the implementation of the recommendations. MAs were trained in performing brown bag reviews and GPs in using online resources and a checklist for systematic medication reviews. The workshop was lead by two of the authors (CJ and JS).
- *Educational material for patients:* The practice teams received posters encouraging patients to take their medication list with them, brown bags as reminders to bring their medication packages to the counseling appointment, and a multilingual “info-tool” for patients on a tablet PC. The brown bags were used due to previous evidence [45]; the info-tool was developed based on a selective literature research and expert consultation to identify relevant learning targets for patients related to medication topics.
- *Implementation action plans:* After the workshop GPs were asked to organize a team meeting and to elaborate a concept of how to implement the recommendations in their individual practice.

Details of the tailored program have been described elsewhere [46].

### Control

GPs in the control group were informed about the general aim of the study and thus about the three evidence-based recommendations we intended to implement. Consequently, they were aware about the “best practice” and the desired professional behavior. As the enrolment of patients into the study included the identification of patients at high risk for ADR, GPs in the control group were also aware of patients in need for intensified care in their practice. Beside this, they did not receive any further instructions or aids for the implementation of the recommendations.

### Primary outcome

The primary outcome was the change in the degree to which the three core recommendations have been implemented into the primary care practice, measured by a set of self-developed indicators (Table 1). The decision for a combined, primary outcome (which had not been validated before) has its rationale in the logic model of the intervention (Fig. 1) and the peculiarities of implementation research: Our aim was to assess whether the implementation of the recommendations increased, i.e., whether the behavior of patients or GPs respectively changed, whereas it was assumed based on prior evidence that a change of behavior would lead to improved health outcomes. Since the recommendations comprised a number of different behavior patterns and since no validated outcomes reflecting the changes in behavior were available, we decided to use a range of indicators reflecting the desired behavior.

**Table 1:** Indicators of successful implementation of the core recommendations (primary outcome)

Recommendation/implementation objective	Indicator		Data source
Recommendation 1: SMC incl. brown bag review was performed at least once	1a	Percentage of patients answering the item “Have you had an appointment for medication counseling with your GP within the last 9 months” in the affirmative	Patient questionnaire
	1b	Percentage of patients answering the item “If yes, have you brought all your medication packages to this appointment” in the affirmative	
Recommendation 2: patients take along medication lists meeting minimum standards	2a	Percentage of medication lists specifying the name of the active substance of each drug	Medication lists generated by the practice
	2b	Percentage of medication lists specifying the reason for prescription for each drug	
	2c	Percentage of medication lists specifying the exact dosage for drugs taken as needed	
	2d	Percentage of medication lists containing instructions for the application of at least one drug	
	2e	Percentage of medication list with a date not older than 9 months	
	3	Percentage of patients with long-term medication having a medication list with them	Oral survey in the practices
Recommendation 3: GPs review the medication systematically using tools to reduce PIM	4a	The response scale of the item “Do you use the PRISCUS list” to review the medication of your patients?” was converted into a percentage value with always = 100%, frequently = 75%, sometimes = 50%, rarely = 25%, never = 0%	GP questionnaires
	4b	The response scale of the item “Do you use the MAI” to review the medication of your patients?” was converted into a percentage value with always = 100%, frequently = 75%, sometimes = 50%, rarely = 25%, never = 0%	

The data sources for the indicators were questionnaires to be completed on a tablet PC in the practice by patients and GPs at baseline and follow-up. Additionally, we planned to analyze the medication lists of the practices and patients. For this purpose, GPs were asked to send the medication lists they had stored in their practice for each patient to the study center at baseline and at follow-up. Furthermore, they were asked to copy and de-identify the medication lists the patients were carrying with them when coming to the practice to complete the questionnaires and send it to the study center. To determine the percentage of patients carrying a medication list with them, we conducted an oral survey in each practice: Each patient entering the practice in a defined time period of 4 h was asked whether he or she was taking long-term medication and whether he or she had a medication list with him or her. The responses were documented anonymously using a tally sheet. Since this survey was conducted at the practice level, the value for the indicator 3 (see Table 1) was identical for GPs working in a group practice.

### Secondary outcomes

A set of questionnaires was used to assess medication-related outcomes at the patient level:

- a self-developed questionnaire assessing the use of medication lists;
- the German Patient Activation Measure (PAM-13D) [47] consisting of 13 item with a mean score ranging from 1–5, higher values reflecting stronger patient activation
- the Medication Adherence Report Scale (MARS) [48] consisting of 5 items with a score ranging from 5 to 25, higher values indicating higher adherence
- the specific part of the German Beliefs About Medicine Questionnaire (BMQ-D) [49] measuring patients’ beliefs about the particular medication prescribed for them. It comprises two sub-scales: the specific necessity scale (SNS) assessing patients’ views on their personal need for their medication and the specific concerns scale (SCS) assessing patients concerns towards their medication. Both scales result in a mean score ranging from 1–5, higher values indication stronger concerns or a stronger belief in the necessity of the medication respectively.
- the PIM prescription rate based on the PRISCUS list was measured using insurance claim data (§300 social code book V).

### Sample size

As specified in the study protocol [41], we had to use a proxy (the PIM prescription rate) for the sample size calculation due to lacking prior knowledge about the primary outcome used in this trial. A total of 40 practices (20 practices per group) were assumed to be sufficient to detect a significant effect of the intervention. Since this sample size was not reached and regarding other limitations of the trial, the findings of this trial should be interpreted carefully.

### Randomization and allocation concealment

We used QC as unit of randomization. Prior to randomization, three conditions for the distribution between intervention and control group were specified:

- The two QCs with the largest number of GPs are not assigned to the same group.
- The number of enrolled patients is approximately equal in both groups.
- The number of GPs is approximately equal in both groups.

Full random sampling was performed using the software “R,” version 3.0.1 [50]. Seven possible solutions meeting the conditions listed above were generated. One solution was selected using a random number function of Microsoft Excel 2010. Randomization was done by researchers of the University Hospital Heidelberg not involved into the trial design after the baseline data collection had been completed, so that allocation concealment was guaranteed.

### Blinding

Due to the nature of the study, GPs and patients could not be blinded to the intervention.

### Statistical methods

The intention-to-treat approach was used for all statistical analyses, meaning that the analysis population included all randomized GPs and patients, which were assigned to the respective treatment group they were originally randomized to, regardless whether they actually received the respective intervention or not.

Socio-demographic data on GPs and patients were analyzed descriptively. Linear regression models were fit to assess the effect of the tailored program on the summary score and each individual indicator. The primary outcome variables at GP level were calculated as the difference between the baseline and the follow-up assessment. Explanatory variables were treatment group and the baseline assessment. Generalized estimating equations were used to adjust for the clustering in practices, with PCPs on level 1 and GPs on level 2.

Further, secondary outcomes at the patient level were fitted using two-level linear mixed models for continuous outcomes, which were fitted using the restricted maximum likelihood method, and two-level generalized mixed models for binary outcomes using the residual pseudo-likelihood method, with practices on level 1 and patients on level 2. All models were adjusted for patient age, gender, highest number of prescribed drugs in one quarter of the year, and number of diagnosed chronic diseases. The score difference between follow-up and baseline was included as dependent variable in the linear mixed models, while intervention group and gender were included as fixed factors, alongside age, highest number of prescribed drugs in one quarter of the year, number of diagnosed chronic diseases and the outcome’s baseline value, which were included as fixed covariates. The binary outcome at follow-up was included as dependent variable in the generalized mixed models, while intervention group, patient gender, and the binary outcome’s baseline value were included as fixed factors, and patient age, highest number of prescribed drugs in one quarter of the year, and number of diagnosed chronic diseases were included as fixed covariate. For all models, type III tests for the intervention group effect were performed, confidence intervals for the effect estimate were calculated, and the ICC was determined.

All statistical tests were two-sided and a significance level of alpha = 0.05 was used in the analysis of the primary outcome. *p* values pertaining to secondary outcomes need to be interpreted descriptively. The models were all fit using PROC GENMOD, PROC MIXED, and PROC GLIMMIX in SAS 9.4 (SAS Institute Inc., Cary, NC).

### Deviations from study protocol

We had to deviate from the study protocol [41] in some points: The concordance of physicians’ and patients’ medication list could not be assessed as intended as we received only very few patient medication lists. The calculated sample size was not reached, which may have resulted in low statistical power. To increase the number of patients we enlarged the included patient population by lowering the minimum age for inclusion from 65 to 50 years*.*

## Results

### Participant flow diagram

**Fig. 2:**
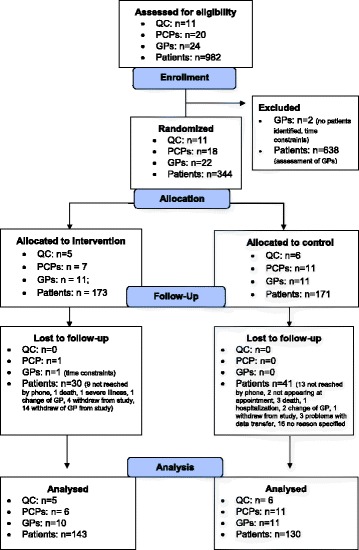
CONSORT flow diagram of the cluster-randomized trial

Figure 2 shows the participant flow diagram of the trial. We invited the moderators of 66 QCs to participate in the study. Twenty-four GPs of 20 practices organized in 11 different QCs agreed to participate. Two GPs were excluded or dropped out respectively before being randomized, because no patients meeting the inclusion criteria could be identified in the practice or because of time constraints, respectively. Thus, 22 GPs from 18 practices were available for randomization.

In total, 982 patients meeting the inclusion criteria were identified using health insurance claims data. Of these, 344 were enrolled into the study after assessment of the treating GPs. Following the procedure described above, 11 GPs from seven practices organized in five different QCs and 173 patients were randomized to the intervention group. Eleven GPs from 11 practices organized in six different QCs and 171 patients were randomized to the control group.

In the intervention group, one GP (having enrolled 14 patients) did not participate in the follow-up-assessment anymore because of time constraints. Another 16 patients of the intervention group were lost to follow-up. Reasons specified were patients not being reached by phone (*n* = 9), death of patient (*n* = 1), severe illness of patients (*n* = 1), change of GP (*n* = 1), and withdrawal from study (*n* = 4). In the control group, 41 patients were lost to follow-up. Reasons specified were patients not being reached by phone (*n* = 13), patients not coming to appointment (*n* = 2), change of GP (*n* = 2), withdrawal from study (*n* = 1), death of patient (*n* = 3), hospitalization of patient (*n* = 1), problems with data transfer (*n* = 3), and reason not specified (*n* = 16). Finally, 273 patients were available for the analysis.

### Recruitment

Recruitment of practices took place from May 2013 to August 2013 and recruitment of patients from September 2013 to December 2013. In the end of January 2014, the intervention started with the workshop and the handing over of the resources to the participants. On 15th of October 2014, the intervention ended with the database closure for documentation of medication counseling. Follow-up data collection of patient data started at the earliest 4 weeks after medication counseling was conducted and ended on 15th of November 2014. Follow-up data collection of GPs data started after the intervention time was completed and ended as well on 15th of November 2014.

### Baseline data

Table 2 shows the characteristics of the participating GPs at baseline. The majority of GPs (82%, *n* = 18) was male and on average 55 years old. These age and gender patterns deviate slightly from a larger, representative survey among German GPs, where a higher percentage of female physicians (35%) was found [51]. There were differences between the intervention and control group concerning the structure of the practice and the sex of the GPs. While the majority of physicians of the intervention group were organized in group practices, all practices of the control group were single practices. None of the physicians of the control group was female. Baseline differences were also present for patient characteristics (Table 3). Patients of the control group were by trend older, received more drugs and suffered from more chronic conditions.

**Table 2:** Socio-demographic data of GPs at baseline

	Total	Intervention	Control
Number of GPs	22	11	11
Number of practices	18	7	11
GPs organized in group practices	8	8	0
Mean Age in years (range; SD)	54.9 (44 – 68; 6.8)	54.2 (44–63; 6.0)	55.6 (44–68; 7.8)
Sex male in % (*n*)	81.8 (18)	63.3 (7)	100 (11)
Mean professional experience as GP in years (range; SD)	22 (8–33; 7.6)	18.8 (8–39; 7.5)	20.1 (11–33; 7.9)
Number of patient contacts per GP per week	222 (120–450; 79.2)	208 (120–450; 94.1)	236 (150–300; 62.2)
Number of MA per practice	4.64 (1–9; SD 2.8)	4.45 (1–9;2.7)	4.82 (1–9; 3.0)

*GP* general practitioner, *SD* standard deviation, *MA* medical assistant

**Table 3:** Socio-demographic data of patients at baseline

	Total	Intervention	Control	*p* value*
Total number of patients	273	143	130	–
Mean Age [years]	72.2 (SD 8.9)	70.8 (SD 9.1)	73.8 (SD 8.38)	0.006
Sex female % (*n*)	55.7 (152)	55.9 (80)	55.4 (72)	0.93
Single % (*n*)	31.7 (85)	30.1 (43)	33.6 (42)	0.54
Living alone % (*n*)	27.7 (74)	24.6 (35)	31.2 (39)	0.23
Not working % (*n*)	87.2 (232)	85.9 (122)	88.7 (110)	0.5
Graduation from high school or university % (*n*)	4.8 (13)	4.9 (7)	4.6 (6)	0.91
Highest number of prescribed drugs in one quarter of the year (range; SD)	7.3 (5–18; 2.6)	7.0 (5–18; 2.6)	7.7 (5–18; 2.6)	0.03
Mean number of diagnosed chronic diseases (range; SD)	5.7 (3–19; 2.8)	5.5 (3–14; 2.2)	6.0 (3–19; 3.2)	0.08

*SD* standard deviation

*p values are not adjusted for multi-level structure and are based on *t* test for continuous and chi-squared test for categorical data

### Outcomes

As depicted in Fig. 2, we analyzed the data of 21 GPs (10 - intervention group, 11 - control group) and 273 patients (130 - intervention group, 143 - control group). Table 4 shows the results on the primary outcome and the various indicators on which it is based. The primary outcome did not differ significantly between the groups. For two indicators, a change in favor of the intervention group was observed: Patients in the intervention group were more likely to have improved receiving structured medication counseling (*p* = 0.017) and a brown bag review (*p* = 0.012) than patients in the control group.

**Table 4:** Results on the various indicators and the summary outcome

Indicator^a^	Baseline T0		Follow-up T1		Treatment effect	*p* value
	Control	Intervention	Control	Intervention		
	MEAN % (SD)	MEAN % (SD)	MEAN % (SD)	MEAN % (SD)	Estimate (95% CI)	
1a	61.8 (27.6)	59.1 (28.6)	49.0 (30.0)	82.7 (20.4)	34.2 (12.4, 55.9)	0.017
1b	43.4 (36.0)	45.7 (37.8)	20.9 (20.9)	59.2 (33.9)	38.8 (15.0, 62.7)	0.012
2a	0 (0)	0.01 (0.02)	4.0 (7.5)	2.6 (6.3)	−3.4 (−8.0, 1.2)	0.18
2b	0 (0)	0.01 (0.02)	0 (0)	1.9 (5.6)	2.1 (−1.4, 5.5)	0.28
2c	1.4 (0.4)	1.7 (0.3)	48.4 (36.7)	25.0 (35.4)	−23.2 (−63.2, 16.8)	0.24
2d	27.4 (43.8)	25.6 (33.3)	31.2 (47.6)	8.2 (22.1)	−21.5 (−50.9, 8.0)	0.18
2e	70.9 (35.0)	55.3 (41.1)	93.9 (12.0)	92.7 (9.2)	0.1 (−9.2, 9.3)	0.99
3	10.9 (5.9)	13.7 (4.7)	20.8 (5.1)	14.5 (19.3)	−8.1 (−15.0, 1.2)	0.08
4a	25.0 (29.6)	15.0 (26.9)	20.5 (18.8)	22.5 (18.5)	5.6 (−5.6, 16.7)	0.35
4b	0 (0)	10.0 (24.2)	2.3 (7.5)	10.0 (21.1)	2.5 (−7.5, 12.4)	0.62
Primary outcome	24.1 (6.7)	22.8 (13.0)	27.9 (6.5)	31.5 (8.0)	4.2 (−0.3, 8.6)	0.10

*SD* standard deviation

^a^For explanation of the indicators, see Table 1

Table 5 shows the results of the analysis on secondary outcomes at the patient level*.* No significant effect on adherence, beliefs in medicine, and patient activation assessed by validated instruments could be shown, neither a significant difference in the PIM prescription rate. Concerning the use of medication lists evaluated by a self-developed patient questionnaire, a significant difference could be observed in one out of nine items: patients in the intervention group reported to show their medication list in the pharmacy more often. There was a tendency that patients in the intervention group were more likely to show their medication list at doctor’s appointments.

**Table 5:** Results of the secondary outcomes

Validated instruments	Baseline T0		Follow-up T1		Estimate (95% CI)	*p* value	ICC
	Control	Intervention	Control	Intervention			
	MEAN (SD)	MEAN (SD)	MEAN (SD)	MEAN (SD)			
MARS score (adherence)	23.3 (2.3)	23.3 (3.7)	23.3 (2.6)	22.3 (3.3)	−1.2 (−2.8, 0.3)	0.11	0.18
PAM score (patient activation)	3.3 (0.4)	3.3 (0.5)	3.3 (0.5)	3.3 (0.5)	0.1 (−0.1, 0.2)	0.48	0.08
BMQ necessity score	4.5 (0.5)	4.2 (0.6)	3.3 (0.5)	3.3 (0.6)	−0.1 (−0.4, 0.3)	0.68	0.20
BMQ concerns score	2.5 (0.9)	2.5 (0.9)	1.4 (0.8)	1.8 (0.9)	0.2 (−0.2, 0.6)	0.24	0.11
Number of PIM prescriptions per year [range]	0.9 (1.8) [0–9]	0.8 (1.8) [0–10]	1.0 (1.9) [0–9]	0.8 (1.8) [0–11]	−0.1 (−0.4, 0.2)	0.37	<0.01
Number of patients with ≥1 PIM prescription per year	32.3% (*n* = 42)	27.7% (*n* = 39)	30.0% (*n* = 39)	26.2% (*n* = 37)	0.9 (0.4, 2.0)	0.81	0.02
Self-developed survey (items with binary response categories)^a^	Control	Intervention	Control	Intervention	Estimate (95% CI)	*p* value	ICC
	% (*n*)	% (*n*)	% (*n*)	% (*n*)			
Do you have a written medication list?	91.5 (118)	91.6 (131)	93.0 (119)	95.8 (137)	1.5 (0.2–12.3)	0.69	0.36
I use my medication list as a reminder	53.4 (62)	60.0 (78)	61.0 (72)	65.0 (89)	1.0 (0.2–4.8)	0.98	0.32
I usually show my medication list at doctor’s appointments	19.8 (23)	42.7 (56)	*22.0 (26)*	*46.0 (63)*	4.7 (0.8–29.6)	0.09	0.39
I usually show my medication list in the pharmacy	0.0 (0)	7.7 (10)	*5.1 (6)*	*28.5 (39)*	12.9 (1.4–117.7)	0.03	0.42
I use my medication list when taking my medicaments	55.2 (64)	45.4 (59)	*63.6 (48.5)*	*59.1 (81)*	0.6 (0.2–2.1)	0.43	0.20
Self-developed survey (items with five-point Likert response scale)^b^	Control	Intervention	Control	Intervention	Estimate (95% CI)	*p* value	ICC
	% (*n*)	% (*n*)	% (*n*)	% (*n*)			
Do you find your medication list comprehensible?	96.6 (114)	93.1 (122)	89.8 (106)	90.9 (130)	1.3 (0.2–10.9)	0.77	0.41
Do you dispose of the old medication list after receiving a new one?	78.0 (92)	69.5 (89)	77.1 (91)	75.9 (107)	0.8 (0.3–1.8)	0.51	0.04
Do you carry your medication list with you (e.g., in your purse?)	33.9 (40)	36.9 (48)	37.3 (44)	48.3 (69)	1.3 (0.4–4.8)	0.64	0.21
Do you note down on your medication list if you take a medicament which you have bought yourself?	13.8 (16)	18.3 (24)	16.9 (20)	25.0 (35)	1.3 (0.4–5.0)	0.65	0.19

*ICC* intracluster correlation coefficient *SD* standard deviation *CI* confidence interval *n* number

^a^Numbers show the percentage of respondents answering the item affirmatively

^b^Numbers show the percentage of respondents answering the items with “always” or “frequently”

### Harms

This study intended to improve professional and patient behaviors regarding the organizational processes in practices. Specifications about the individual treatment of patients were not made. Thus, no additional harms for patients had to be expected and were not reported to us.

## Discussion

This study examined the effect of a tailored program on the primary and secondary outcomes, which reflected the degree of implementation of three recommendations for patients with multimorbidity and polypharmacy. We did not observe a significant difference in changes between groups and the treatment effects for the single indicators were heterogenous: Our results suggest that the implementation of structured medication counseling and brown bag reviews may have improved in the intervention group and small effects may be present on patients’ self-reported use of medication lists when buying drugs in pharmacies. On the contrary, some of the indicators for medication list use by GPs had—though not significant—a reverse tendency indicating a higher adoption of the recommendations in the control group.

Other tailored programs focusing on polypharmacy used similar strategies than those we selected for our program, such as academic detailing, education, treatment algorithms, patient information leaflets and paper bags [45, 52]. We did not include pharmacists, a frequently used strategy with heterogeneous effects [53], because this is difficult to implement in German primary care as collaboration of practices and pharmacists is not well established. Systematic reviews on interventions to improve polypharmacy often conclude that the effects of the interventions are conflicting and variable [53–57], so it remains unclear under which circumstances which strategy effectively improves the use of polypharmacy.

The fact that patients in the intervention group were more likely to receive medication counseling and a brown bag review during the previous 9 months than those in the control group suggests that the TI effectively induces changes on practice organization and related determinants. However, long-term implementation has to be examined. There are different possible reasons why the intervention had no or only a minor effect on the other two recommendations: The baseline performance for some outcomes was comparatively high, such as 90% of the patients having a medication list. In other German studies, this rate was lower between 25 to 50% [17] indicating that there might have been a selection bias of practices and/or patients, maybe due to the fact that all practices participated in quality circles, where pharmaceutical issues are regularly discussed. The PIM prescription rate of 26–32%, however, was comparable to larger studies [12, 13]. Furthermore, it may be possible that important implementation factors were not successfully addressed by the program, either because they were not identified or because the selected strategies were not effective. A comprehensive process evaluation which was conducted additionally to the main analysis to examine this issue, showed that fidelity to some of the intervention components was low and that some important barriers were not or insufficiently modified and deduced suggestions for improvement of the intervention (Jager C, Steinhauser J, Freund T, Kuse S, Szecsenyi J, Wensing M. A tailored program to implement recommendations for multimorbid patients with polypharmacy in primary care practices – process evaluation of a cluster-randomised trial. Under review in Implementation Science). Other possible reasons for the low effectiveness of the trial might be related to limitations of the trial.

### Limitations

A limitation of the trial is that the sample size was lower than planned, which may have led to lack of statistical power to detect intervention effects. On the other hand, we had to use a proxy measure for the power calculation which implies uncertainties regarding this calculation. Furthermore, we had to use a range of non-validated outcome parameters since no validated measures for the behavior change intended to be induced by the intervention were available. So it is unclear, whether the used outcome parameters reliably measure implementation improvement.

The baseline differences in patient characteristics (patients of the control group were by trend older, received more drugs and suffered from more chronic diseases) and the differences in practice structure (all GPs of the control group worked in single practices while GPs of the intervention group worked in group practices) might be relevant determinants for the implementation of guideline recommendations, especially since the ICC was extraordinarily high for several outcomes, suggesting that patient outcomes strongly depended on the practice with a high variance between practices.

Although we reduced the minimum age for inclusion to 50 years, the patterns for age, sex, family status, and living arrangements were similar to those of a German representative epidemiological study on multimorbidity [58]. Previous research found that 25% of patients suffering from at least 6 chronic conditions are younger than 50 years [59]. This indicates that polypharmacy is not only a challenge in elderly patients but that also younger patients might profit from intensified care. This should be taken into account in further research projects.

Given the limitations of the study, the findings of the trial need to be interpreted carefully in an explorative manner. However, we believe that the information provided by the study is nevertheless useful for other researchers in the field of medication management—a relatively young research field in Germany which is receiving more and more scientific and political attention.

## Conclusions

This study examined a tailored program intending to improve the implementation of three evidence-based recommendations for the management of multimorbid patients with polypharmacy in primary care. No effect on the combined primary outcome could be substantiated. Yet, the results suggest that the program may lead to improved implementation of structured medication counseling and brown bag reviews, while only a marginal effect on the use of medication lists and no effect on the use of tools for medication reviews could be observed. Due to limitations of the trial, the results should be interpreted in an explorative manner. The factors facilitating and hindering successful implementation are examined ﻿separately in a comprehensive process evaluation.

## Abbreviations

ADR: Adverse drug reaction
BMQ-D: German version of the “Beliefs in Medicine Questionnaire”
GP: General practitioner
HZV: “hausarztzentrierte Versorgung” (GP-centered care contract)
ICC: Intraclass correlation coefficient
MA: Medical assistant
MAI: Medication appropriateness index
MARS: Medication Adherence Report Scale
PAM-13D: German version of the “Patient Activation Measure”
PIM: Potentially inappropriate medication
PomP: “Implementation of recommendations for polypharmacy in multimorbid patients”
QC: Quality circles
SCS: Specific Concerns Scale (of the BMQ-D)
SMC: Structured medication counseling
SNS: Specific Necessity Scale (of the BMQ-D)
TICD: “Tailoring Interventions for Chronic Diseases” project
